# The Treatment of Aneurism

**Published:** 1896-02-08

**Authors:** A. Pearce Gould

**Affiliations:** Senior Assistant Surgeon to the Middlesex Hospital.


### THE TREATMENT OF ANEURISM.

By A. PEARCE GOULD, F.R.C.S., Senior Assistant Surgeon to the Middlesex Hospital.

UNLESS there is some urgent condition calling for immediate interference, even external aneurism should be treated by careful rest in bed and elevation of the part before resorting to any further step. In some cases a carefully-applied bandage to the limb is of use. With these simple measures recovery not very unfrequently occurs. Where it is not successful the surgeon has to choose between several alternatives. For external aneurism the choice generally lies between some form of ligature and some form of compression of the main artery. When it was first employed, and for a long time afterwards, compression had one great advantage over the treatment by ligature in that it was unattended with the risk of wound-infection, which often led not only to local inflammation and suppuration, but also to general blood poisoning and to secondary hæmorrhage. With the perfecting of antiseptic surgery the special dangers of the ligature have been minimised, and it is necessary to reconsider the opinions formerly arrived at. Compression is troublesome, in some cases difficult to carry out efficiently, and in all cases involves more or less severe pain to the patient. Its results are not so certain as those of ligature, and if it is tried for a long time and fails it lessens the chance of success of the ligature, owing to its causing enlargement of the anastomosing arteries. The ligature can now, with aseptic surgical methods, be employed with the best prospect of success, with the minimum of risk to the individual, and hardly any suffering. These are conditions which tell powerfully in favour of the ligature. Where time is an object it is certainly better to resort at once to the ligature, but in other cases compression may be tried, if it is applied carefully, with not too much force, and, especially, if the compression is not kept up long. The best form of compression is digital compression. The finger is superior to every form of artery compressor for three chief reasons. The contact of skin with skin is less injurious than the contact of any other substance with skin. The pressure of the finger can be more exactly regulated to the exact and varying conditions of each individual case than can any mere mechanical compressor, and the pressure of the finger can be limited to the artery alone in a way that is impossible when using a compressor. The many skilled assistants it requires is its one serious drawback, and except in hospitals where relays of dressers can be readily obtained, digital compression is not often available.

In view of the great safety of the ligature, treatment by compression should not be made very painful or trying to the patient, and therefore it should not be prolonged. The best plan is to give it one good trial by continuous pressure kept up so as to arrest all pulsation in the aneurism for several hours, and, if necessary, morphia or an anæsthetic may be given. If this leads to consolidation of the sac, gentle pressure on the artery must be continued for two days afterwards in order to protect the recently-formed clot from the disintegrating effects of the forcible blood streams. If no marked effect is produced, treatment by ligature should be resorted to.

A special form of compression was introduced by Dr. Reid, and it has generally been spoken of as Reid's treatment. He employs Esmarch's elastic bandage, applied from the distal extremity of the limb up to the aneurism, then, missing this, it is continued around the limb for a short distance above the aneurism. By this means the aneurism and the artery from which it springs are kept full of stagnant blood. By the entire stasis that it causes, this form of treatment differs from all other varieties of compression, and it has met with a large measure of success. The elastic bandage has to be left on for an hour or an hour and a half, and an anæsthetic is usually necessary for most of this time. When it is removed some form of compression should be applied to the main artery on the cardiac side, so that any clot that has formed may be protected. The cases best adapted for this treatment are popliteal aneurisms of small or medium size, which are not growing rapidly and have firm sacs. Employed in well-chosen cases, Reid's treatment has been very successful, and it has the merit of acting quickly, and of producing its effect more rapidly than any other form of compression.

As an adjunct to any form of compression, it may be well to combine the administration of some drug known to possess a hastening influence upon the coagulation of the blood. This would be more imperative if it was found beforehand that the patient's blood did coagulate unusually slowly. The substances which we know to possess this power are chloride of calcium, carbonic acid gas, and solutions of nucleo-albumen. Of these, the best for our purpose would be the chloride of calcium, and it should be given in doses of twenty grains once or even twice a day, for a day or two before as well as during the treatment by compression.

With reference to the ligature of an artery in its continuity we must content ourselves with a brief statement of the most important practical points. In the first place, the surgeon should choose for the seat of the ligature a spot in the diseased artery on the cardiac side of the aneurism, as near to the aneurism as is consistent with the performance of the operation without injury to the sac, provided that the vessel can be exposed without unusual difficulty. Stress has been laid upon the advantage of tying an artery at some distance above an aneurism, inasmuch as it is more likely to be healthy than those closed to the sac. But this has been shown not to be the case. It was also thought that it was an advantage to have a large branch intervene between the ligature and the aneurism, but this point, too, is now known to be unimportant. Indeed, it has been shown that in the majority of cases the artery becomes obliterated from the seat of ligature to beyond the aneurism, and from this fact it follows that the closer the ligature is to the aneurism the shorter the length of artery obliterated, and the less the interference with the circulation in the part. In applying this rule, the surgeon must attach great importance to preserving the integrity of the sac, and must also design his operation so as to expose the artery readily, easily, and without the division of other important structures.

